# Smartphone-Based and Data-Driven Superstructure State Prediction Method for Highway Bridges in Service

**DOI:** 10.3390/s22155620

**Published:** 2022-07-27

**Authors:** Jixin Duan, Weili He, Shizhan Xu, Zhaoyuan Zhong, Liang Huang

**Affiliations:** 1 School of Civil Engineering, Zhengzhou University, Zhengzhou 450001, China; 202022212014159@gs.zzu.edu.cn (J.D.); 202022212014168@gs.zzu.edu.cn (W.H.); xushizhan@zzu.edu.cn (S.X.); 2 Institute of Longspan Bridge Monitoring and Control, Zhengzhou University, Zhengzhou 450001, China; 3 School of Water Conservancy Engineering, Zhengzhou University, Zhengzhou 450001, China; 202112222014362@gs.zzu.edu.cn

**Keywords:** Cox, survival analysis, smartphone, bridge superstructure, life prediction

## Abstract

Survival analysis is a data-driven approach that is widely used in various fields of biomedical prognostic research, and it is highly reliable in the processing of time-event data. This study developed a method for evaluating the service performance of bridge superstructures using the built-in acceleration sensor of smartphones and the prediction of survival analysis theory. It will be used to assist in the daily maintenance and repair of small and medium bridges. The effects of the upper load-bearing structure, upper general structure, bearings, deck paving, expansion joints, and frequency ratio on the deterioration of the bridge superstructure were investigated. The results show that the first-order vibration frequency of the bridge can be effectively detected by the built-in acceleration sensor of the mobile phone, but its low sensitivity and high output noise make it impossible to accurately detect the higher-order frequencies of the bridge. The upper load-bearing members, the upper general structure, the bearing, the deck pavement, and the frequency ratio are all related to the changing trend of the technical condition level of the bridge superstructure.

## 1. Introduction

With the continuous acceleration of infrastructure construction, the transportation industry has developed by leaps and bounds. However, with the continuous growth of transportation capacity, the traffic density and vehicle load on highways at all levels are also increasing, which makes a considerable number of existing highway bridges unable to meet the needs of transportation [1,2]. In particular, the deterioration of a large number of small- and medium-span bridges is particularly prominent. Accurate prediction of highway bridge superstructure performance is critical for effective maintenance and repair, as superstructures such as bridge decks are a major concern and budget factor for transportation agencies [3,4]. Therefore, it is imminent to carry out safety status assessments and real-time health monitoring of existing bridge structures, especially small- and medium-span bridge structures. At present, most of the assessments and early warnings of the safety status of bridge structures are carried out by installing sensors and other monitoring equipment on the bridge structure to conduct long-term real-time monitoring of the bridge operation status and related physical quantities [5,6]. However, the objects of long-term health monitoring of bridge structures are mostly large, and large bridges, as well as medium and small span bridges with unique shapes and important positions. There are few related studies on the health monitoring of common medium and small span bridges with a large number of bases. In addition, the existence of censored data is a common data problem in bridge data samples, which can affect bridge degradation analysis [7]. Survival analysis theory has been widely used in medicine, pharmacy, patient prognosis, economics, and other fields because it can process censored data and does not require many assumptions about the form of survival function [8,9,10,11,12]. Therefore, survival analysis has been gradually applied to the deterioration analysis of the service performance of engineering structures in recent years [13,14]. However, the data sources for the survival analysis of existing bridge structures are mainly assessed by visual inspection by bridge inspectors, which leads to a great deal of subjectivity.

At present, the most widely used bridge performance degradation prediction methods mainly include deterministic methods and random methods. The deterministic methods gradually reduce the attention of researchers because they cannot reflect the randomness in the bridge operation process [15]. The probabilistic method of bridge performance degradation prediction is mainly to simulate the degradation process of each component of the bridge over time by establishing different forms of probability density functions, such as establishing a continuous-time Markov process degradation model of concrete highway bridges [16,17], established a semi-Markovian process based on Weibull distribution to simulate the degradation process of urban bridges [18], established a concrete highway bridge deck deterioration model based on Bayesian survival theory to explore the impact Factors in Bridge Deck Performance [4]. Stevens et al. [19] proposed a new application of survival analysis based on visual inspection of sparse data based on four types of data: bridge construction type, function, number of spans, and road class. However, because these data are too superficial, it is difficult to reflect the different degradation trends of various components of the bridge, and there is a large bias caused by subjective influence. Therefore, these methods cannot make an accurate assessment of the deterioration of bridge structures.

With the continuous development of smartphones, sensor technology has been widely used in mobile phones. Due to the continuous improvement of sensor performance, in-depth research has been carried out in indoor positioning [20], user behavior recognition [21,22], and scenario simulation [23] in recent years. In addition, sensors such as accelerometers embedded in smartphones can also be used to detect the smoothness of the road surface and identify the modal characteristics of bridges [24,25,26]. Therefore, the fundamental frequency of the bridge can be quickly detected by smartphones arranged at key sections of the bridge structure and introduced into the bridge survival analysis as a research factor. Liu et al. [27] used two Android mobile phones and the SPAN-IGM-A1 inertial integrated navigation system to conduct a comparative experiment on the acceleration signal and vibration detection of Xuzhou Han Bridge, which verifies the feasibility of portable and low-cost Android smartphones for bridge vibration detection. Zhao et al. [28] carried out experiments such as the stay cable force test and bridge vibration test to prove that smartphones can accurately measure the natural frequency of the first mode of bridge structures, but it is difficult to obtain the natural frequencies of higher-order modes. Elhattab et al. [29] effectively improved the sensitivity of smartphone accelerometers by exploiting the phenomenon of stochastic resonance. The feasibility of their proposed SR filter is verified by comparing the bridge vibration characteristics measured by an iPhone device with a high-sensitivity wireless sensor network consisting of 15 accelerometers. In summary, the built-in acceleration sensor in smartphones is feasible in bridge structure vibration signal acquisition and bridge health monitoring and has great advantages due to its large number of devices and simple operation.

To analyze the effects of many factors on survival outcomes and survival time at the same time, a multivariate analysis method is required. However, traditional multivariate analysis methods are not applicable, cannot deal with both survival outcomes and survival time, and cannot take full advantage of the incomplete information provided by the censored time [15]. In addition, most of the research factors considered in the existing bridge performance prediction models are based on appearance inspection and environmental factors, and the accuracy of the obtained data is largely affected by human factors.

**Figure 1:**
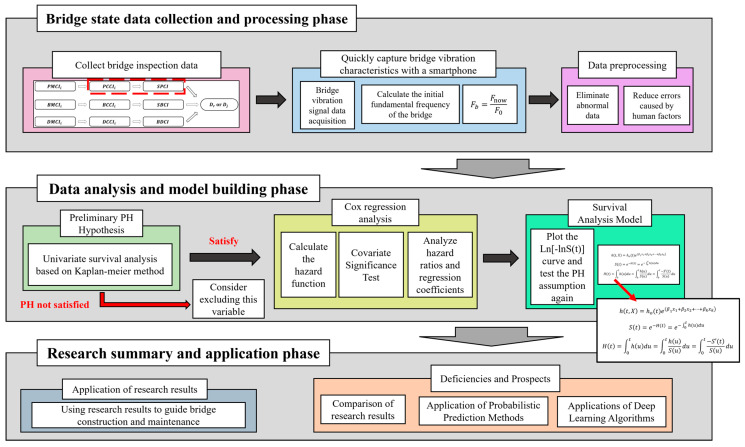
Overview of research methods.

Considering that it is unrealistic to do real-time health monitoring for a large number of small- and medium-span bridges. This study developed a method for evaluating the service performance of bridge superstructures using the built-in acceleration sensor of smartphones and the prediction of survival analysis theory. It will be used to assist in the daily maintenance and repair of small and medium bridges. The effects of upper load-bearing structure (PCCIa), upper general structure (PCCIb), bearings (PCCIc), deck paving (DMCIa), expansion joints (DMCIb), and frequency ratio (the ratio of the current frequency to the initial frequency of the bridge superstructure, which is named Fb in this article) on the deterioration of the bridge, superstructures were investigated. Firstly, the measurement structure of the built-in accelerometer of the smartphone is compared with the measurement structure of Donghua’s accelerometer in the field, which further verifies the effectiveness of the built-in accelerometer of the mobile phone to detect the natural frequency of the bridge. Secondly, the initial vibration frequency of the bridge is calculated by the finite element modeling and the formulas in the relevant codes. Finally, all the collected research factors are brought into the survival analysis model, and the influence of each research factor on the deterioration of the bridge superstructure in different periods is calculated. The research protocol is shown in Figure 1.

## 2. Experimental Verifications

### 2.1. Methodology for Android Phone Sensors

To allow developers to quickly access the sensor and easily read the raw data collected by the sensor, the Android SDK provides developers with a very convenient open interface, which enables developers to access the sensors to obtain the required data. Android sensor framework development mainly includes the following four categories:

(1) Sensor Manager: Users are allowed to access the built-in sensor class of the device by creating an instance of this class, obtaining the list of sensors in the device, registering event listeners, and logging out event listeners when the program exits. Various sensor constants such as sensor accuracy and set sampling frequency can be realized by Sensor Manager.
(2) Sensor: Users are allowed to create a specific instance of a sensor, through which the user can obtain a specific sensor type.
(3) Sensor Event: It can be used to create an object of a sensor event and obtain event information related to the sensor, such as sensor type, timestamp, raw data, and data accuracy.
(4) Sensor Event Listener: It is used to create a callback method. When the data collected by the sensor or the accuracy of the sensor changes, two callback functions can be used to help developers obtain data and interrupt through callbacks. During the development of Android sensors, the sensor must be registered through Sensor Manager and the sampling frequency should be set. The actual sampling frequency is related to the specific software and hardware configuration of the mobile phone.

The short-time Fourier transform (STFT) is a general tool for speech signal processing. It defines a very useful class of time and frequency distributions, where the complex magnitude of an arbitrary signal is specified as a function of time and frequency [30]. In practice, the process of computing the short-time Fourier transform is that the longer-time signal is divided into shorter segments of the same length, and the Fourier transform is performed on each of the shorter segments. The basic idea is that a long-term non-stationary stochastic process is regarded as the superposition of a series of short-term stochastic stationary signals, and the short-term nature can be realized by adding a window function in time (that is, intercepting a part of the source data). With this approach, whatever frequency component is found, it must occur within the specified period in which the signal is being intercepted, avoiding recording an incorrectly long frequency band and affecting the entire spectrogram. The specific implementation method is to multiply the window function and the source signal in the Fourier transform to realize the addition of the window and translation in the vicinity, and then perform the Fourier transform [31].

The definition of the short-time Fourier transform is given in Equation (1).

(1) $$STFT_{x}\left(t,f\right)=\int_{-\infty }^{+\infty }x\left(\tau \right)h\left(\tau -t\right)e^{-i2\pi ft}d\tau$$

where x(τ) is the input signal, and $STFT_{x}\left(t,f\right)$ is the short-time Fourier transform of the signal x(τ).

The center E{h(t)} and radius Δ{h} of the window function h(t) are shown in Equations (2) and (3), respectively. The width of the window function h(t) is 2Δ{h}.

(2) $$E\left\{h\left(t\right)\right\}=\frac{\int_{-\infty }^{+\infty }t{\left|h\left(t\right)\right|}^{2}dt}{{\left||h|\right|}_{2}^{2}}$$

(3) $$\Delta \left\{h\right\}=\sqrt{\frac{\int_{-\infty }^{+\infty }{\left(t-E{\left\{h\right\}}^{2}\right)}^{2}{\left|h\left(t\right)\right|}^{2}dt}{{\left||h|\right|}_{2}^{2}}}$$

**Figure 2:**
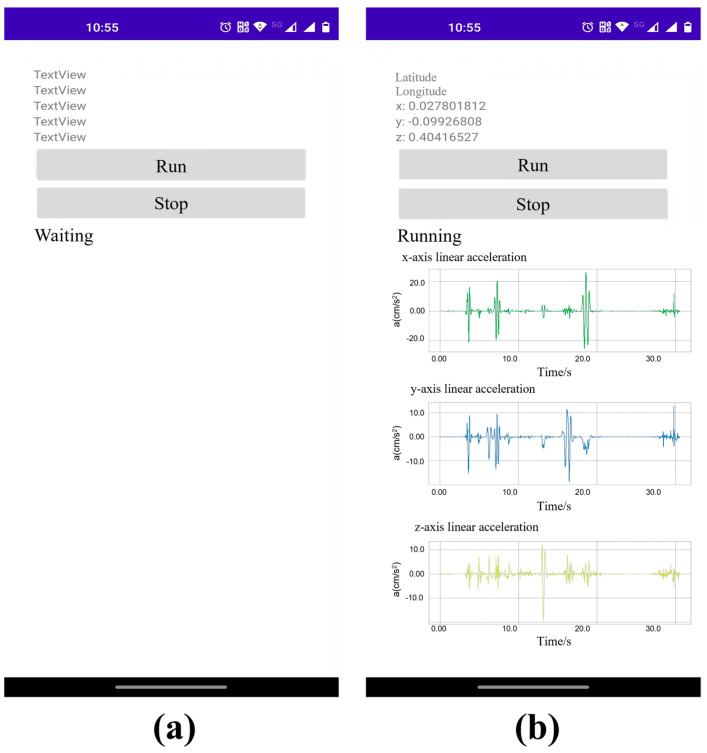
Mobile phone acceleration acquisition application: (**a**) acceleration test waiting interface; (**b**) acceleration acquisition interface.

The interface of the mobile phone acceleration acquisition program is shown in Figure 2.

In general, the analytical performance of the short-time Fourier transform can be judged by the shape and area of the time–frequency window rectangle. The shape of the time–frequency window is fixed, and the smaller the window area is, the stronger the time–frequency analysis capability of the short-time Fourier transform is, otherwise it is poor. 

The inverse of the short-time Fourier transform is shown in Equation (4):

(4) $$x\left(\tau \right)=\int_{-\infty }^{+\infty }\int_{-\infty }^{+\infty }STFT_{x}\left(t,f\right)h\left(\tau -t\right)e^{i2\pi ft}dfdt$$

It can be seen that the analysis performance of the short-time Fourier transform depends to a large extent on whether the selected window function is reasonable, and the selection of the appropriate window function can effectively improve the time–frequency analysis performance of the short-time Fourier transform.

When performing a short-time Fourier transform analysis on a signal, on the one hand, the length of the selected window function should be as short as possible, which can improve the time precision of the short-time Fourier transform. On the other hand, the time width of the selected window function should be as long as possible, which can improve the frequency accuracy of the short-time Fourier transform. Therefore, in this experiment, the original data of 500 s is selected, the time course of 10 s is used as a band, the interval of 5 s is used as the selection interval, and 0–10, 5–15, 10–20, 12–25… are selected in turn. The method of overlapping panes to select experimental data can effectively avoid inaccurate results caused by human errors and acquisition errors, and reduce experimental errors.

### 2.2. Experimental Specimens

To verify the reliability of the mobile phone accelerometer on simply supported girder bridges and continuous girder bridges, Zhengzhou Xiaoliu Bridge and Nancun Yellow River Bridge are selected for the experiment. The specific information about these two bridges is shown in Table 1.

### 2.3. Experimental Conditions and Setup

In this experiment, the pulsation method is used to measure the vibration characteristics of the bridge to test the accuracy of the measurement results of the mobile phone accelerometer. The equipment used in the experiment is shown in Table 2.

**Figure 3:**
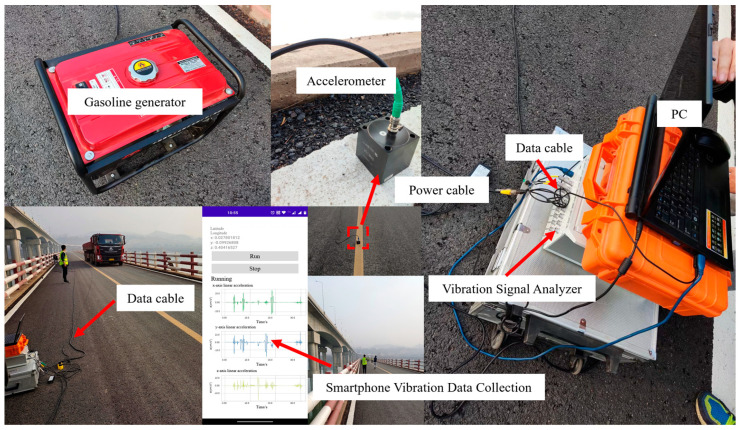
Experiment site layout.

As shown in Figure 3, the field experimental setup consisted of a dynamic signal analyzer, six accelerometers, a signal processor, several data transmission lines, and three smartphones with vibration data collection software installed.

Through the preliminary finite element calculation results, it can be seen that the significant positions of the free vibration deformation of the simply supported girder bridge are mainly at the third and fourth points of the test span. At the same time, the significant positions of free vibration deformation of continuous girder bridges are mainly distributed in the quarter point section of the test span. To effectively detect the dynamic characteristics of the bridge, the acceleration sensor and the smartphone were arranged at the 1/4 L, 1/2 L, and 3/4 L sections of the test span.

First, six accelerometers (DH610V) were placed at 1/4 L, 1/2 L, and 3/4 L on both sides of the bridge with the same span, and were connected to the dynamic signal analyzer (DH8302) through data cables. Then, place the three mobile phones in the middle of the bridge deck at the 1/4 L, 1/2 L, and 3/4 L sections, respectively. Finally, the vibration data from the corresponding locations are measured using vehicle excitation and artificial excitation.

### 2.4. Experimental Results

**Figure 4:**
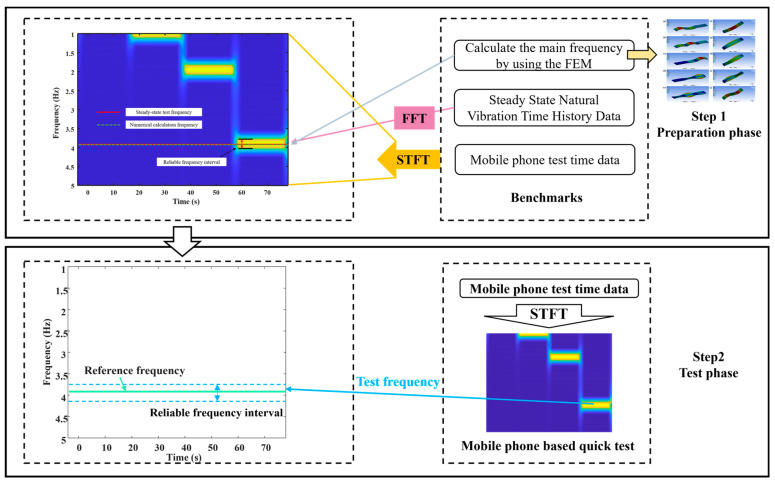
Vibration signal acquisition principle.

First, the finite element calculation frequency and the bridge natural vibration frequency obtained by professional test equipment are compared and calibrated, and the bridge natural vibration frequency obtained from the test is used as the reference frequency. Then, a short-time Fourier transform is performed on the mobile phone test data to obtain a time–frequency map. Several significant frequency intervals can be obtained from the time–frequency diagram (see the bright area in Figure 4), and the upper and lower limits of the bright area where the reference frequency is located are used as the frequency confidence interval. Finally, the mobile phone sensor is used to quickly test to obtain the time history data of the bridge. A new time–frequency map can be obtained by performing a short-time Fourier transform on the time-history data. Select the bright area that falls within the frequency confidence interval, and take the middle value as the test frequency.

**Figure 5:**
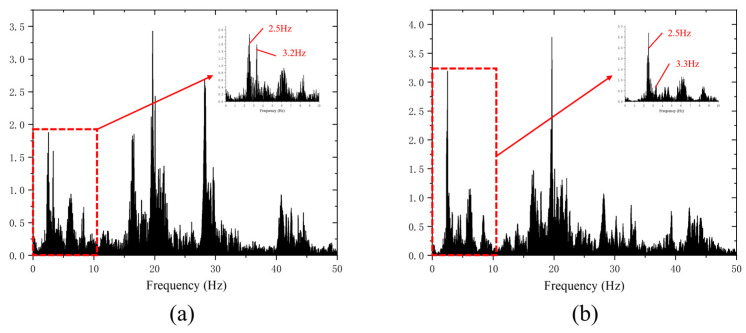
Nancun Yellow River Bridge natural vibration frequency test results: (**a**) accelerometer vibration test results; (**b**) vibration test results for smartphones.

According to the bridge design data and site survey investigation, ANSYS finite element analysis software is used to establish a model to calculate the dynamic characteristics of the structure. The calculated span of the simply supported beam is 50 m, and the calculated first six-order frequency values of the bridge are 2.92, 3.16, 8.15, 10.29, 10.57 and 17.37 Hz, respectively. In addition, the accelerometer and smartphone field vibration test results are shown in Figure 5. Figure 5a clearly shows the high precision and low noise of the traditional accelerometer test, which can clearly analyze the natural frequency of the bridge. However, the built-in accelerometers in smartphones are flawed in the identification of high-order frequencies due to the problem of noise acquisition. Therefore, only the first-order frequency of the smartphone test is considered to be introduced into the subsequent survival analysis as the base frequency.

## 3. Deterioration Prediction of Superstructure

In this study, the Cox proportional hazards regression model was used to consider the influence of various influencing factors on the deterioration of the bridge superstructure. The Cox proportional hazards model is the most popular mathematical modeling method for estimating survival curves when multiple explanatory variables are considered simultaneously, and it can be used to model the time to a specific event based on the value of a given covariate [32]. The Cox proportional hazards regression model is shown in Equation (5).

(5) $$h_{i}\left(t,x\right)=h_{0}\left(t\right)\exp(\beta_{1}x_{i1}+\beta_{2}x_{i2}+\cdots +\beta_{n}x_{in})$$

where $h_{i}\left(t,x\right)$ = the hazard rate for the ith case at time t; $h_{o}\left(t\right)$ = baseline hazard at time t; *n* = number of covariates; $\beta_{n}$ = value of the nth regression coefficient; and $x_{in}$ = value of the ith case of the nth covariate.

It can be seen from Equation (5) that the index part is a parametric model and the benchmark risk function $h_{0}\left(t\right)$ is a non-parametric model because it can use different distribution models according to different data due to its uncertainty. Therefore, the Cox model is a semi-parametric model. The condition for the establishment of the Cox model is that each covariate satisfies the proportional hazards assumption (PH). The hazard ratio (HR) for the two individuals is shown in Equation (6).

(6) $$HR=\frac{h_{i}\left(t,x\right)}{h_{j}\left(t,x\right)}=\frac{h_{0}\left(t\right)\exp\left(\beta_{1}x_{i1}+\beta_{2}x_{i2}+\cdots +\beta_{n}x_{in}\right)}{h_{0}\left(t\right)\exp\left(\beta_{1}x_{j1}+\beta_{2}x_{j2}+\cdots +\beta_{n}x_{jn}\right)}=\exp\left(\overset{n}{\underset{i,j=1}{\sum }}\beta_{i}\left(X_{in}-X_{jn}\right)\right)$$

From Equation (6), it can be seen that the hazard ratio is independent of time t, and the hazard ratio is a constant, which means that the covariate regression coefficient is fixed. In addition, $\beta_{i}>0$ leads to a hazard ratio HR>1, which means that it increases the risk of the outcome event. Similarly, $\beta_{i}<0$ leads to the HR<1, which reduces the risk of the outcome event [3,7,33]. The calculation of the regression coefficients of the covariates requires a partial likelihood function, which is estimated using the maximum likelihood. The partial likelihood function is shown in Equation (7).

(7) $$L_{c}\left(\beta \right)=\overset{n}{\underset{i=1}{\prod }}q_{i}=\overset{n}{\underset{i:E_{i}=1}{\prod }}\frac{\exp\left(\beta_{1}X_{i1}+\beta_{2}X_{i2}+\cdots +\beta_{n}X_{in}\right)}{\sum_{s\in R\left(T_{i}\right)}\exp\left(\beta_{1}X_{s1}+\beta_{2}X_{s2}+\cdots +\beta_{n}X_{sn}\right)}$$

where the values $T_{i}$ and $E_{i}$ are the respective event time and event indicator for the ith observation. The equation is defined over the set of bridges with an observable event $E_{i}=1$. The risk set $R\left(T_{i}\right)=\left\{i:T_{i}\right\}$ is the set of bridges still at risk of failure at time t [34].

Define f(t) as the probability density function of bridge superstructure degradation. The probability of degradation of the bridge superstructure at time t can be expressed as F(t). The specific form of F(t) is shown in Equation (8). Therefore, the survival probability of the bridge superstructure at time t (the probability that the bridge superstructure still maintains the original state level) can be obtained by 1−F(t). The specific form of the survival function is shown in Equation (9).

(8) $$F\left(t\right)=P\left(T\ll t\right)=\int_{0}^{t}f\left(u\right)du$$

(9) $$S\left(t\right)=P\left(T>t\right)=1-F\left(t\right)=\int_{t}^{\infty }f\left(u\right)du$$

h(t) in Equation (5) is defined as the conditional probability of death of the bridge superstructure at time t, which can also be expressed as Equation (10).

(10) $$h\left(t\right)=\underset{\Delta t\to 0}{\lim}\frac{P\left(t<T<t+\Delta t|T>t\right)}{\Delta t}=\frac{f\left(t\right)}{S\left(t\right)}=\frac{-S^{\prime }\left(t\right)}{S\left(t\right)}$$

Integrating both sides of Equation (10) yields Equation (11).

(11) $$S\left(t\right)=e^{-H\left(t\right)}=e^{-\int_{0}^{t}h\left(u\right)du}$$

### 3.1. Research Factors

Bridge stiffness changes due to material deterioration, cracks, and other diseases. The reduction in bridge stiffness directly leads to a decrease in its natural frequency. Therefore, it can be considered to obtain the fundamental frequency of the bridge through the vibration test, analyze the change law of the inherent fundamental frequency and the overall stiffness of the bridge, and then understand the actual operating state of the bridge [35]. Therefore, to evaluate and predict the actual operating state of the bridge as comprehensively as possible, we consider introducing the fundamental frequency of the bridge into the prediction model.

**Figure 6:**
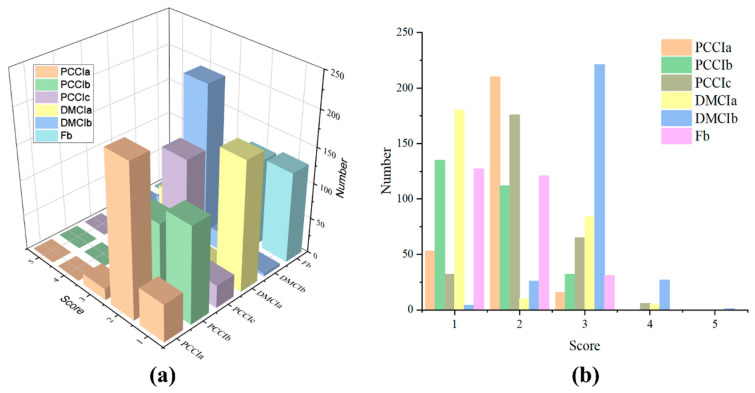
Distribution of technical status of bridge components: (**a**) Overall distribution of bridge component status grades; (**b**) Number of bridges in different state levels.

In addition to the bridge dynamic parameters collected by the mobile phone acceleration sensor, this paper also uses the inspection records of 279 highway bridges stored in the bridge periodic inspection database in a certain province to predict and model the service performance of highway bridges. The bridges collected in this paper are all less than 20 years old. More than 90% of the bridge’s upper load-bearing structure, upper general components, bearings, bridge deck pavement, and expansion joints are grades 1–3, and almost no bridge components are grade 5. The technical state-level distribution of each component of the bridge is shown in Figure 6. 

According to the author’s previous research results, it can be found that the decay speed of the bridge deck system, superstructure, and substructure levels is accelerated in turn [33]. We consider the performance decay prediction based on the technical status of the bridge superstructure and further explore the influence of each component of the bridge superstructure on the service performance of the bridge [16]. However, since the deck system of a girder bridge mainly includes deck pavement, expansion joint devices, sidewalks, railings and guardrails, drainage systems, lighting and signs, and other components, most of these components have little impact on the operational safety of the bridge structure. Considering that the weight of bridge deck pavement and expansion joint device in the comprehensive evaluation of the whole bridge is 8% and 5%, respectively [36], and their quality has a significant impact on the driving comfort. Therefore, we decided to use the evaluation grades of bridge decking and expansion joint devices as research factors in this paper.

### 3.2. Data Preprocessing

The technical condition grades of bridges are divided according to the provisions in the “Standards for Technical Conditions Evaluation of Highway Bridges” [36]. See Table 3 for details. Therefore, the upper load-bearing structure class, the upper general structure class, the bearing class, the deck pavement class, and the expansion joint class are regarded as multivalued ordered independent variables.

The initial base frequency of the bridge should be calculated by the finite element method. For the following conventional structures, when there is no more precise method to calculate, the following formula can also be used to estimate:

(1) Simply supported girder bridge

(12) $$f_{1}=\frac{\pi }{2l^{2}}\sqrt{\frac{EI_{c}}{m_{c}}}$$

In Equation (12), l refers to the calculated span of the structure, m; *E* refers to the elastic modulus of the structural material, Pa; $I_{c}$ refers to the moment of inertia of the mid-span section of the structure, $m^{4}$; $m_{c}$ refers to the mass per unit length at the midspan of the structure, kg/m.

(2) Continuous girder bridge

(13) $$f_{1}=\frac{13.616}{2\pi l^{2}}\sqrt{\frac{EI_{c}}{m_{c}}}$$

(14) $$f_{2}=\frac{23.651}{2\pi l^{2}}\sqrt{\frac{EI_{c}}{m_{c}}}$$

In Equations (13) and (14), when calculating the positive bending moment effect and shear force effect caused by the impact force of the continuous beam, the fundamental frequency $f_{1}$ is used; when calculating the negative bending moment caused by the impact force of the continuous beam, the fundamental frequency $f_{2}$ is used.

Different bridge structure types and spans have different fundamental frequencies of bridge structures. To measure the change of the fundamental frequency of the bridge uniformly, we propose the frequency ratio $F_{b}$ as a unified indicator to characterize the change of the fundamental frequency of the bridge. The calculation formula of the frequency ratio index is shown in Equation (15).

(15) $$F_{b}=\frac{F_{N}}{F_{0}}$$

In Equation (15), $F_{N}$ refers to the current fundamental frequency of the bridge, Hz; $F_{0}$ refers to the initial fundamental frequency after the bridge is built, Hz. In addition, it can be seen from Equation (5) that $F_{b}\leq 1$, the better the bridge state, the closer $F_{b}$ is to 1.

The frequency ratio is graded concerning the relevant content on the delineation of the technical condition grade boundaries of bridges in the “Standards for Technical Conditions Evaluation of Highway Bridges” [36]. The change of the natural frequency of the bridge gradually slows down with the extension of time. To accurately describe the change of the bridge frequency ratio index in the early stage of the bridge construction, the limit range of the bridge frequency ratio grade gradually becomes larger with the increase in the grade. The specific classification method is shown in Table 4. After the frequency ratio rank is divided, the frequency ratio rank can be introduced into the survival analysis model as a multivalued ordered independent variable.

Bridge inspection data may have data missing, reporting errors, and data errors caused by human factor observation. Therefore, it is necessary to preprocess the data before introducing the data to ensure the accuracy of the prediction model results. This paper mainly carries out the following data processing steps. First, delete the record that the technical condition level of the whole bridge has dropped by more than 2 in the adjacent two years [15]. The reason is that in this case, the bridge may have design defects, the bridge is located in a relatively harsh environment, or it has been damaged by human factors. Then, correct the human subjective bias in the detection process. By examining the data collected this time, it was found that some bridges were rated better than the previous ones. However, the bridge did not undergo repairs during this period. Further inspection found that the reason was that different engineers had different subjective scores for the same bridge at the level boundary of technical conditions. Therefore, the detection records of the bridges at the technical condition grade dividing line are screened out, and then the two technical condition grades of the bridge are treated as equal, that is, the event is regarded as not occurring.

### 3.3. PH Assumption Verification

To verify whether the upper load-bearing members, upper general members, supports, bridge deck paving, expansion joints, and frequency ratio conform to the PH assumption, and to understand the influence of these factors on the bridge superstructure grade, the Kaplan–Meier method was used to analyze the above several influencing factors draw survival curves.

If the univariate survival curves are disjoint or substantially disjoint, it indicates that the corresponding variables can be introduced into the Cox survival analysis. The survival curve drawn by the Kaplan–Meier method is shown in Figure 6.

**Figure 7:**
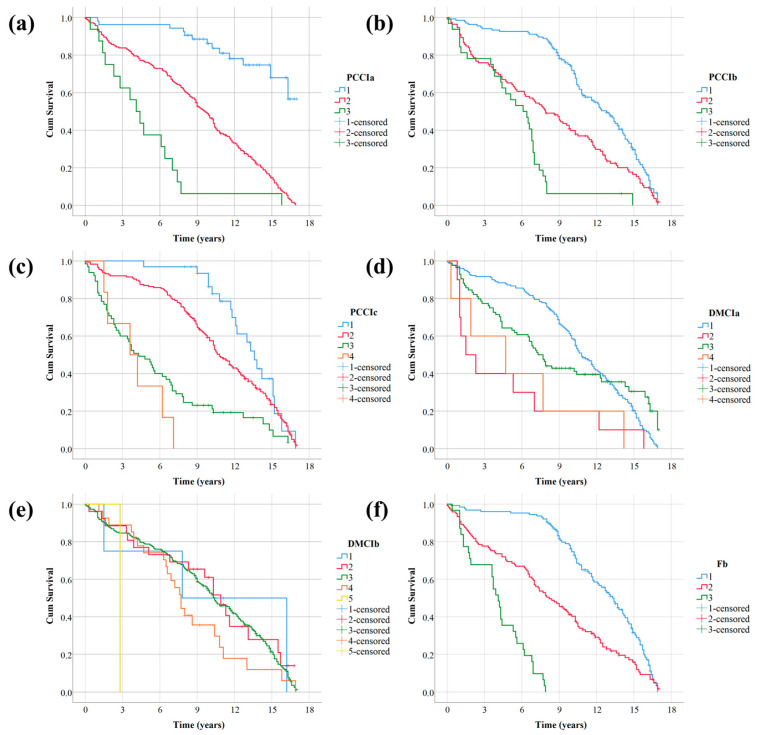
Univariate survival curve: (**a**) survival curve of PCCIa; (**b**) survival curve of PCCIb; (**c**) survival curve of PCCIc; (**d**) survival curve of DMCIa; (**e**) survival curve of DMCIb; (**f**) survival curve of Fb.

It can be seen from Figure 7 that, except for the level of expansion joints, the survival curves of the other research factors basically do not cross. It can be preliminarily determined that PCCIa, PCCIb, PCCIc, DMCIa, and Fb basically meet the PH assumption, and can be introduced into the following Cox survival analysis model as research factors.

As can be seen from Table 5, except for expansion joints, the significance tests of Log Rank, Breslow, and Tarone-Ware for the remaining five variables are all less than 0.005. This further indicates that PCCIa, PCCIb, PCCIc, DMCIa, and Fb are statistically significant and can be used in the Cox multivariate survival analysis model. The significance of all three tests for expansion joints is greater than 0.05, which means that the expansion joint rating is not statistically significant and should be removed from the model.

However, expansion joints will be damaged due to changes in external temperature difference, thermal expansion, contraction of concrete, and bridge deflection caused by various loads, bridge deck longitudinal slope, and driving braking force [37,38]. Then, the joints fall off or the cast-in-place concrete surface is partially peeled off or even damaged. This will not only cause bumps when vehicles go down the bridge, but also reduce the overall service level of the bridge, and even affect the safety of the bridge [39]. Damage to expansion joints can lead to water seepage. Water seepage not only erodes the beam body but also corrodes the support, which affects the normal shrinkage of the beam body. In the end, the stress on the related structure of the beam body is much larger than the design stress, which affects the overall structural safety of the bridge. Therefore, the expansion joint was still introduced into the multivariate Cox survival analysis model as a research factor.

### 3.4. Cox Survival Analysis

PCCIa, PCCIb, PCCIc, DMCIa, DMCIb, and Fb were introduced into the model as multivalued ordered independent variables. The independent variables are entered into the equation according to the probability of the score test and then removed according to the partial likelihood ratio test. Since PCCIa, PCCIb, PCCIc, DMCIa, DMCIb, and Fb all have more than three levels in the bridge data collected this time, this variable is transformed into a dummy variable and introduced into the model. The regression coefficient (B) of each variable, the standard error (SE) of B, its Wald test significance value, the significance value of the coefficient, and the average marginal effect are shown in Table 6. If significance < 0.05, then the researcher concludes that the covariate is useful to the model. Positive regression coefficients mean the covariate increases hazard, whereas negative coefficients correspond to reduced hazard. The hazard ratio (HR) for a given covariate appears as Exp(B) in the output, which is also the predicted change in the hazard for a unit increase in the predictor [40].

It can be seen from Table 6 that the upper load-bearing structure, the upper general structure, the bearing, the deck pavement, and the frequency ratio are the important factors affecting the survival time of the bridge superstructure. The *p*-values for the expansion joint test were all greater than 0.05, indicating that it was not statistically significant and was excluded from the model. The data in column B of the table represent the regression coefficients of each covariate. It can be seen that the regression coefficients of all independent variables in the equation are positive, which indicates that the risk of degrading the bridge superstructure class increases with the increase in the superstructure, upper general members, bearings, and deck pavement, and frequency ratio class. In addition, the influence of frequency ratio, upper general members, bridge deck pavement, bearings, and upper load-bearing members on the service performance of bridge superstructure gradually increases. Exp(B) in Table 6 represents the downgrade risk of a bridge with a certain component level relative to the superstructure level of a bridge with a corresponding component level of 1 when other variables are equal. For example, the degradation risk of bridge superstructure grade with $PCCIa_{2}$ is 8.132-fold that of $PCCIa_{1}$, and the degradation risk of bridge superstructure grade with $PCCIa_{3}$ is 13.089-fold that of $PCCIa_{1}$.

The risk function derived from the maximum likelihood estimation results in Table 6 is shown in Equation (16).

(16) $$\begin{array}{cc} h\left(t\right)=h_{0}\left(t\right)\exp & (2.096\times PCCIa_{2}+2.572\times PCCIa_{3}+0.628\times PCCIb_{2}\\ & +1.350\times PCCIb_{3}+0.486\times PCCIc_{2}+1.490\times PCCIc_{3}\\ & +2.074\times PCCIc_{4}+0.402\times DMCIa_{2}+0.491\times DMCIa_{3}\\ & +1.710\times DMCIa_{4}+0.415\times Fb_{2}+0.808\times Fb_{3}) \end{array}$$

The linear combination part of the variables on the right side of the risk function expression is proportional to the risk function. The larger the value, the greater the risk, reflecting the prognosis of an individual, which is called the prognostic index (PI). The prognostic index of this example is shown in Equation (17).

(17) $$\begin{array}{cc} PI=2.096\times PCCIa_{2} & +2.572\times PCCIa_{3}+0.628\times PCCIb_{2}+1.350\times PCCIb_{3}\\ & +0.486\times PCCIc_{2}+1.490\times PCCIc_{3}+2.074\times PCCIc_{4}\\ & +0.402\times DMCIa_{2}+0.491\times DMCIa_{3}+1.710\times DMCIa_{4}\\ & +0.415\times Fb_{2}+0.808\times Fb_{3} \end{array}$$

**Figure 8:**
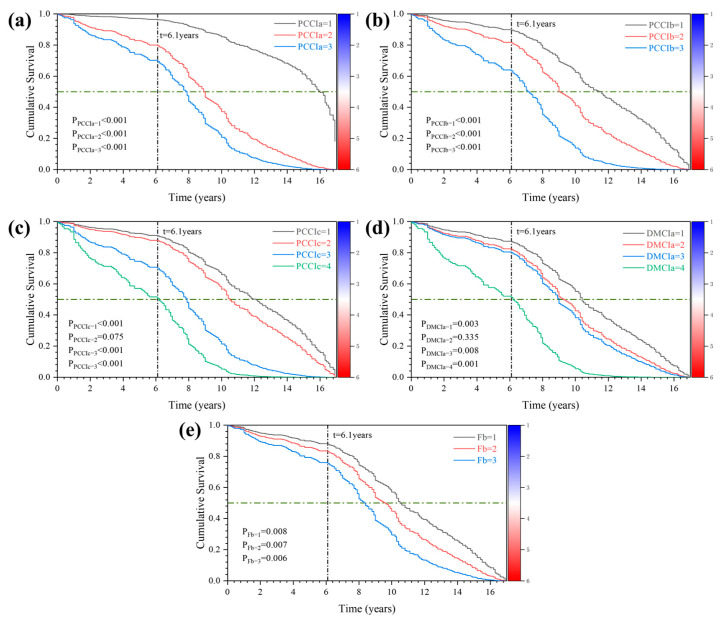
Cox survival curve: (**a**) Cox survival curve of PCCIa; (**b**) Cox survival curve of PCCIb; (**c**) Cox survival curve of PCCIc; (**d**) Cox survival curve of DMCIa; (**e**) Cox survival curve of Fb.

Through the relationship between the risk function and the survival function of the bridge superstructure, the survival function curve of each influencing factor can be obtained. The Cox survival function plot is shown in Figure 8. As can be seen from Figure 8a, the rate of deterioration of the bridge superstructure began to accelerate in the sixth year. The median superstructure survival times for bridges with superstructure classes 1, 2, and 3 were 16, 7.8, and 8.9 years, respectively, which implies that the decay rate of the superstructure gradually decreased over time. In the initial stage of bridge construction, the mid-span deflection is mainly caused by the shrinkage and creep of concrete. With the extension of the service time of the bridge, the shrinkage and creep effect of the concrete gradually slows down, and the deflection of the superstructure caused by it also gradually decreases. However, with the prolongation of service time, the corrosion of steel bars, cracks of beams and slabs, and spalling of concrete voids gradually appear, which will have a very adverse effect on the superstructure of bridges and accelerate the increase in mid-span deflection. In addition, the median survival times of deck pavement classes 1, 2, 3, and 4 were 10.3, 9.2, 9, and 6.2 years, respectively, indicating that the effect of decking on the superstructure varies with time gradually increases. With the prolongation of bridge service time, the bridge deck pavement will appear cracks, damaged voids, and other phenomena. The continuous deterioration of bridge deck pavement causes the bridge superstructure to receive a greater level of dynamic load, which will accelerate the damage to bridge pavement. In addition, the continuous deterioration of the bridge deck pavement greatly reduces driving comfort. The significant reduction in driving comfort will adversely affect the driver’s future route selection, which in turn will gradually reduce the traffic volume on the route.

**Figure 9:**
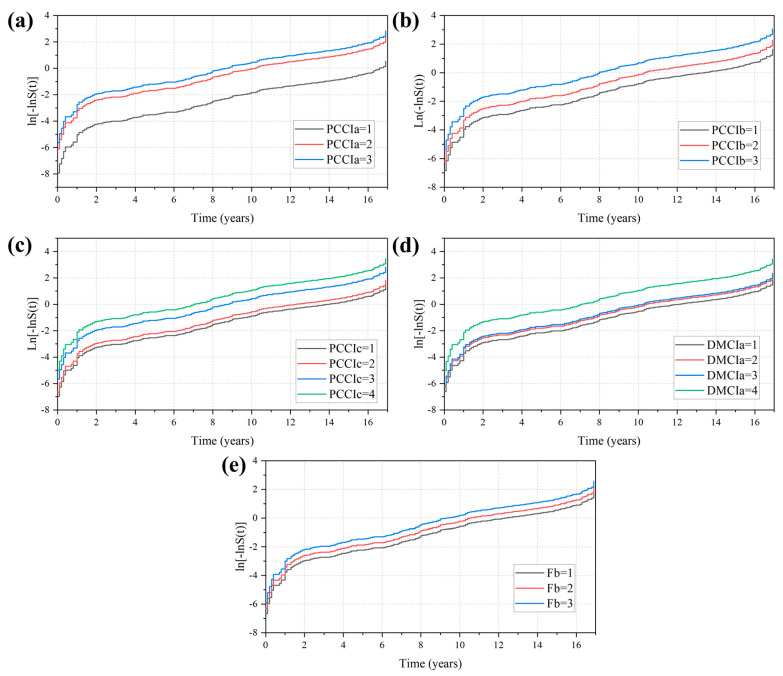
PH Assumption verification: (**a**) Ln[−LnS(t)]-T curve of PCCIa; (**b**) Ln[−LnS(t)-T curve of PCCIb; (**c**) Ln[−LnS(t)]-T curve of PCCIc; (**d**) Ln[−LnS(t)]-T curve of DMCIa; (**e**) Ln[−LnS(t)]-T curve of Fb.

Finally, the model is tested for the PH assumption through the relationship between the survival function and the hazard function (see Equation (11) for details), and a plot of Ln[−LnS(t)]-Time is plotted in Figure 9. Except for the expansion joints, the parallel curves of the different state levels of the components prove that they meet the requirements of the PH assumption.

The expansion joints in this study did not meet the significance test and were not included in the model, but it did not mean that the expansion joints did not affect the use state of the superstructure [37]. On the one hand, the bridge survival data collected this time did not classify the bearing types, and different bearing forms may have different effects on the degradation of the bridge superstructure. On the other hand, expansion joints will be more or less blocked and stuck no matter what state the bridge is in as a whole. Expansion joints are attachments that are subjected to maximum dynamic loads during bridge use. The small unevenness of the bridge deck will make it bear a large impact force, which can easily cause damage to the expansion joint. When the expansion joint device is damaged to a certain extent, it will cause the bridge deck to jump, thus affecting the structural safety of the bridge. Therefore, the bridge expansion joint is very important to the overall structure of the bridge.

## 4. Conclusions

With the continuous increase in service time, the service performance of bridges will gradually decay at different rates. Accurately predicting the decay trend of bridge service performance is of great significance for the safe operation and maintenance of bridges. Considering that it is impossible to arrange real-time sensors on thousands of small and medium bridges to monitor structural safety, this study developed a method to analyze the fundamental frequency of bridges by collecting dynamic parameters of bridges with smartphone sensors. 

Bridge fundamental frequency and routine inspection data were used as research factors to analyze the deterioration trend of the superstructure of bridges in service. The variation characteristics of the upper load-bearing member, upper general structure, bearing, bridge deck pavement, expansion joint, and bridge fundamental frequency with time under different technical condition levels were investigated. The following main conclusions were obtained.

The built-in acceleration sensor of an Android smartphone can effectively collect the first-order vibration frequency of the bridge, but its low sensitivity and high output noise level make it impossible to directly measure the higher-order vibration frequency of the bridge.

The upper load-bearing members, the upper general structure, the bearing, the deck pavement, and the frequency ratio are all related to the changing trend of the technical condition level of the bridge superstructure. The median survival period of the superstructure of the bridge with the upper load-bearing member grade 1 is 16 years, which is much larger than that of the bridge with the higher load-bearing member grade. The research results are consistent with the actual deterioration trend of the bridge superstructure. The bridge superstructure degradation prediction method based on survival analysis and bridge fundamental frequency makes full use of the information provided by incomplete bridge survival data. This not only improves the prediction accuracy of the model but also reduces the artificial subjective influence caused by the visual inspection data to a certain extent. The research has a certain guiding significance for the decision-making of the bridge maintenance management department.

In this paper, the rapid collection of bridge frequencies by smartphone is combined with survival analysis theory to study the decay law of bridge superstructure over time, but the comparison with other bridge service performance prediction models is not considered. In addition, prediction methods based on machine learning and deep learning theory are gradually being applied to the prediction of structural life in recent years. However, due to the small amount of bridge health detection data, the above methods cannot be more efficiently applied in bridge service performance prediction. In the future, the author plans to infer the general decay law of bridge structures through a large amount of data analysis, combine hyperparameter optimization to generate a large number of bridge survival simulation data, and then apply it to the prediction model based on deep learning theory.

## Figures and Tables

**Table 1:** Basic Information of Experimental Bridges.

Bridge Name	Superstructure	Pier and Foundation	Span (m)	Length (m)	Width (m)
Xiaoliu Bridge	Simply supported beam	Double pile pier	35	252	25
Nancun Yellow River Bridge	Continuous beam	Double pile pier rectangular hollow pier	50	1456	7.5

**Table 2:** Experimental Equipment.

Equipment	Specifications	Quantity	Purpose
Dynamic Signal Analyzer	DH8302	1	Signal processing
Accelerometer	DH610V	6	Data collection
Signal processor	HP	1	Process and store data
Data transmission line	/	6	Transfer data
Redmi	K40	1	Data collection
Huawei	P30	1	Data collection
Motorola	Edge X30	1	Data collection

**Table 3:** Bridge Technical Condition Classification Limit.

Technical Status	$\mathrm{Technical}\;\mathrm{Status}\;\mathrm{Level}\;D_{j}$				
	1	2	3	4	5
$D_{r}$	[95, 100)	[80, 95)	[60, 80)	[40, 60)	[0, 40)
Condition	Well/Good	Better	Normal/Poor	Poor	Danger

**Table 4:** Frequency Ratio Classification Limit.

Frequency Ratio Level		
1	2	3
[95, 100]	[80, 95)	[0, 80)

**Table 5:** Chi-Square and Significance Tests.

Covariate	Index	Log Rank	Breslow	Tarone-Ware
PCCIa	Chi-Square	67.491	56.001	62.714
	Sig.	<0.001	<0.001	<0.001
PCCIb	Chi-Square	59.299	71.599	68.905
	Sig.	<0.001	<0.001	<0.001
PCCIc	Chi-Square	63.976	86.470	79.177
	Sig.	<0.001	<0.001	<0.001
DMCIa	Chi-Square	13.089	28.334	20.699
	Sig.	0.004	<0.001	<0.001
DMCIb	Chi-Square	7.904	7.009	7.950
	Sig.	0.095	0.135	0.093
Fb	Chi-Square	119.583	118.336	121.984
	Sig.	<0.001	<0.001	<0.001

**Table 6:** Regression Coefficients and Significance Tests.

Covariate	B	SE	Wald	Sig.	Exp(B)	Lower	Upper
$PCCIa_{1}$			48.772	0.000			
$PCCIa_{2}$	2.096	0.309	46.127	0.000	8.132	4.442	14.889
$PCCIa_{3}$	2.572	0.452	32.360	0.000	13.089	5.396	31.748
$PCCIb_{1}$			28.872	0.000			
$PCCIb_{2}$	0.628	0.152	16.993	0.000	1.874	1.390	2.527
$PCCIb_{3}$	1.350	0.279	23.478	0.000	3.857	2.234	6.658
$PCCIc_{1}$			43.641	0.000			
$PCCIc_{2}$	0.486	0.273	3.174	0.075	1.625	0.953	2.773
$PCCIc_{3}$	1.490	0.304	24.086	0.000	4.439	2.448	8.050
$PCCIc_{4}$	2.074	0.510	16.547	0.000	7.593	2.928	21.600
$DMCIa_{1}$			17.179	0.003			
$DMCIa_{2}$	0.402	0.417	0.931	0.335	1.495	0.660	3.386
$DMCIa_{3}$	0.491	0.184	7.086	0.008	1.633	1.138	2.344
$DMCIa_{4}$	1.710	0.493	12.051	0.001	5.529	2.105	14.518
$Fb_{1}$			9.880	0.008			
$Fb_{2}$	0.415	0.154	7.274	0.007	1.515	1.120	2.048
$Fb_{3}$	0.808	0.297	7.417	0.006	2.243	1.254	4.012
$DMCIb_{1}$				0.373			
$DMCIb_{2}$				0.276			
$DMCIb_{3}$				0.204			
$DMCIb_{4}$				0.248			
$DMCIb_{5}$				0.339			

Note: $PCCIa_{1}$, represents bridges with superstructure class 1, and similarly $PCCIa_{2}$, and $PCCIa_{3}$, represent bridges with superstructure classes 2 and 3, respectively. PCCIb, PCCIc, DMCIa, DMCIb, and Fb have the same meanings as above.

## Data Availability

Some or all data, models, or code generated or used during this study are proprietary or confidential and may only be provided with restrictions.
